# Primary Metabolite Profile Changes in *Coffea* spp. Promoted by Single and Combined Exposure to Drought and Elevated CO_2_ Concentration

**DOI:** 10.3390/metabo11070427

**Published:** 2021-06-29

**Authors:** Ana M. Rodrigues, Tiago Jorge, Sonia Osorio, Delphine M. Pott, Fernando C. Lidon, Fábio M. DaMatta, Isabel Marques, Ana I. Ribeiro-Barros, José C. Ramalho, Carla António

**Affiliations:** 1 Plant Metabolomics Laboratory, Instituto de Tecnologia Química e Biológica António Xavier, Universidade Nova de Lisboa (ITQB NOVA), 2780-157 Oeiras, Portugal; amrodrigues@itqb.unl.pt (A.M.R.); tiago.jorge89@gmail.com (T.J.); 2 Instituto de Hortofruticultura Subtropical y Mediterránea “La Mayora”, Departamento de Biología Molecular y Bioquímica, Universidad de Málaga—Consejo Superior de Investigaciones Científicas (IHSM-UMA-CSIC), 29071 Málaga, Spain; sosorio@uma.es (S.O.); dpott@uma.es (D.M.P.); 3 GeoBioSciences, GeoTechnologies and GeoEngineering (GeoBioTec), Faculdade de Ciências e Tecnologia (FCT), Universidade NOVA de Lisboa (UNL), 2829-516 Monte de Caparica, Portugal; fjl@fct.unl.pt; 4 Departamento de Biologia Vegetal, Universidade Federal Viçosa (UFV), Viçosa 36570-090, Brazil; fdamatta@ufv.br; 5 Plant Stress & Biodiversity Lab, Centro de Estudos Florestais (CEF), Instituto Superior Agronomia (ISA), Universidade de Lisboa (ULisboa), Tapada da Ajuda, 1349-017 Lisboa, Portugal; isabelmarques@isa.ulisboa.pt

**Keywords:** climate change, *Coffea arabica*, *Coffea canephora*, coffee tree, elevated CO_2_, GC-TOF-MS, mass spectrometry, plant metabolomics, water deficit

## Abstract

Climate change scenarios pose major threats to many crops worldwide, including coffee. We explored the primary metabolite responses in two *Coffea* genotypes, *C. canephora* cv. Conilon Clone 153 and *C. arabica* cv. Icatu, grown at normal (aCO_2_) or elevated (eCO_2_) CO_2_ concentrations of 380 or 700 ppm, respectively, under well-watered (WW), moderate (MWD), or severe (SWD) water deficit conditions, in order to assess coffee responses to drought and how eCO_2_ can influence such responses. Primary metabolites were analyzed with a gas chromatography time-of-flight mass spectrometry metabolomics platform (GC-TOF-MS). A total of 48 primary metabolites were identified in both genotypes (23 amino acids and derivatives, 10 organic acids, 11 sugars, and 4 other metabolites), with differences recorded in both genotypes. Increased metabolite levels were observed in CL153 plants under single and combined conditions of aCO_2_ and drought (MWD and SWD), as opposed to the observed decreased levels under eCO_2_ in both drought conditions. In contrast, Icatu showed minor differences under MWD, and increased levels (especially amino acids) only under SWD at both CO_2_ concentration conditions, although with a tendency towards greater increases under eCO_2_. Altogether, CL153 demonstrated large impact under MWD, and seemed not to benefit from eCO_2_ in either MWD and SWD, in contrast with Icatu.

## 1. Introduction

Coffee is an important agricultural commodity worldwide that provides income and employment to millions of people in developing countries. Coffee is cultivated in over 80 countries throughout the tropics with ca. 99% of the production based on *Coffea arabica* L. (Arabica coffee) and *Coffea canephora* Pierre ex Froehner (Robusta coffee) [1,2]. Estimated global climate change scenarios will impose adverse environmental conditions with deep impacts on crops, mainly due to water constraints and heat stress [3]. In this context, the coffee sector is expected to face serious challenges in the upcoming decades as several studies have demonstrated the climate change sensitivity of coffee species, particularly *C. arabica*, with impacts on suitable cultivation areas, yield, and bean quality, as well as increased pest and disease incidence and economic losses [4,5,6,7,8,9].

One of the major components of global climate change is an increase in air CO_2_ concentration, coupled with rising air temperatures (with the worst estimate pointing to a global average increase of up to 936 ppm CO_2_ and 4.5 °C by 2100), together with more frequent and prolonged drought events associated with unpredictable rainfall patterns [10]. Such events are expected to threaten the sustainability of agricultural production on a global scale and be responsible for severe constraints to plant growth and productivity [11]. Nevertheless, there are interesting and potentially positive effects of elevated CO_2_ concentration (eCO_2_) on several crop plants, usually associated with increased photosynthetic activity [12,13]. That is the case for coffee, under adequate water availability [14,15,16,17], which displays greater C-investment in reproductive structures [18], ultimately increasing productivity [1]. Furthermore, eCO_2_ was recently reported to increase coffee plant resilience to heat stress [19,20,21], while preserving leaf mineral balance [22] and bean quality [23]. Additionally, although the future positive impact of eCO_2_ on plants subject to water deficits can be species-dependent [24], studies reported that eCO_2_ can clearly reduce drought impact on plant photosynthesis, growth, and yields [25,26,27]). These effects were reported to occur in *Coffea* spp. as well, where eCO_2_ mitigated the negative impacts of drought on photosynthetic performance and components [28,29,30,31,32].

Under field conditions, plants are often exposed to a combination of different environmental changes. In this case, the plant response to combined changing conditions should be regarded not as a sum of each one applied independently, but as a unique response that can reflect a positive (i.e., increased tolerance) or negative (i.e., susceptibility) interaction between them [33,34,35]. Abiotic stresses can deeply impact plant metabolism and cause a reconfiguration of the metabolic network in order to allow for the maintenance of metabolic homeostasis and the production of compounds that can mitigate the effects of stress [36,37]. Drought stress is one of the major threats to agriculture, since it constrains a wide number of physiological, morphological, and biochemical processes, impacting growth, nutrient uptake, C-assimilation, and partitioning, and ultimately yield and quality [28,38,39]. Hence, it is essential to understand the plant response to this critical adverse environmental condition. Metabolic adjustments in response to drought include the regulation of photosynthesis and the maintenance of cell osmotic potential through the accumulation of metabolites such as phytohormone abscisic acid and osmolytes, including amino acids, soluble sugars, the raffinose family of oligosaccharides (RFOs), polyols, and polyamines [36,40,41,42,43]. Osmolyte accumulation in response to abiotic stress conditions has an important role in the maintenance of cell turgor—via a decrease in the osmotic potential of the cell—and protection against oxidative damage by a decrease in the levels of reactive oxygen species (ROS) to restore cellular redox balance [43]. Moreover, stress-tolerant plants usually show higher levels of some of these stress-related metabolites, even under normal growth conditions, to keep their metabolism prepared for adverse environmental conditions [41].

Complementing the previous studies of our team, and regarding the coffee plant response to single and combined exposure to drought and/or eCO_2_ at the physiological, biochemical, and molecular level [28,29,32,44], here we further explored the changes in gas chromatography time-of-flight mass spectrometry (GC-TOF-MS) primary metabolite profiling in plants exposed to increasing water deficit severity, and how eCO_2_ might influence drought response and plant resilience in *Coffea* spp. To date, GC-MS is the most widely accepted analytical technology used in post-genomic plant metabolomics studies [43,45,46,47]. Genotypes from the two main coffee producing species, *C. canephora* cv. Conilon Clone 153 (CL153) and *C. arabica* cv. Icatu (Icatu), were grown under normal atmospheric (aCO_2_) or elevated (eCO_2_) air CO_2_ concentration, coupled with either well-watered conditions (WW), or moderate (MWD) or severe (SWD) water deficit conditions. Our findings provide important and timely evidence regarding the role of eCO_2_ in the response to drought in these species, contributing to improvements in knowledge regarding coffee plant performance in the face of ongoing and future global climate changes.

## 2. Results

**Figure 1:**
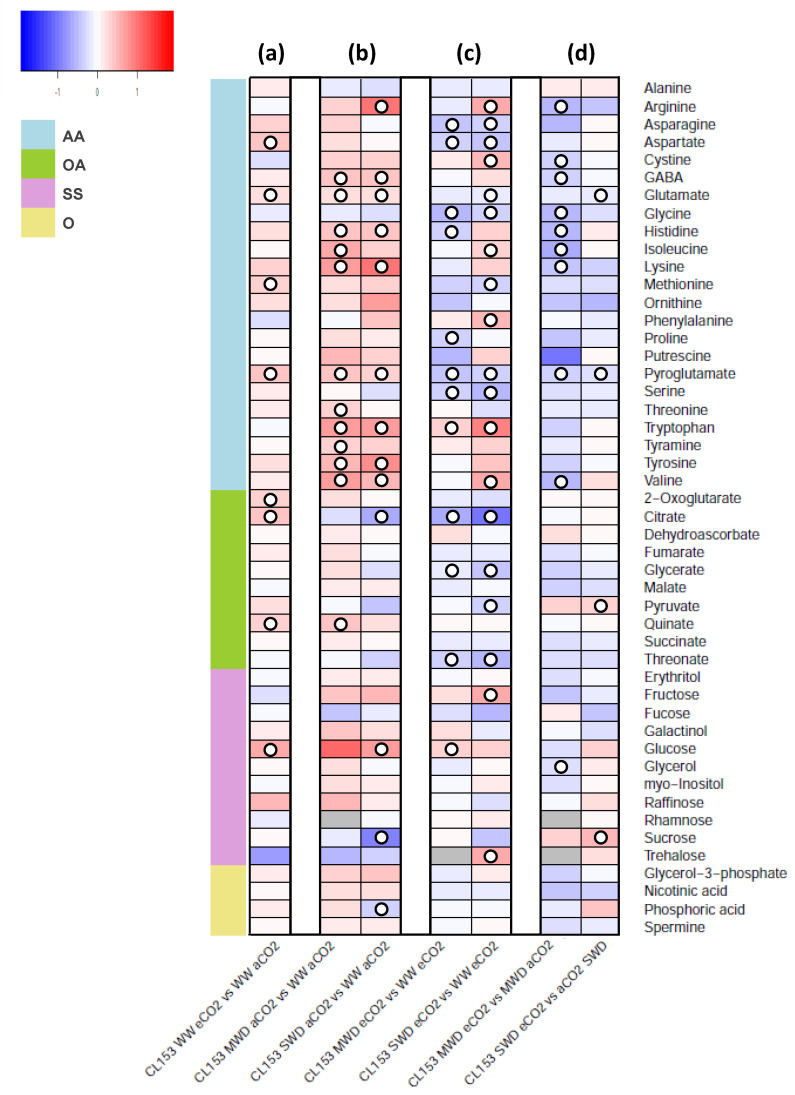
Heatmap representing the changes in relative levels of primary metabolites in leaves of *C. canephora* cv. Conilon Clone 153 (CL153), grown under 380 (aCO_2_) or 700 (eCO_2_) ppm CO_2_, and three water availability levels (WW, well-watered; MWD, moderate water deficit; SWD, severe water deficit). Results indicate (**a**) single exposure to eCO_2_, (**b**) single exposure to each water deficit level under aCO_2_, (**c**) exposure to each water deficit level under eCO_2_, and (**d**) eCO_2_ effect within each water deficit level. Relative values (as means of three to six independent measurements) were normalized to the internal standard (ribitol) and the dry weight of the samples; false color imaging was performed on log_10_-transformed GC-TOF-MS metabolite data. Significant changes using one-way ANOVA are indicated as (○) for *p* < 0.05. Heatmaps and clustering were performed in R software [48,49] using the “gplots” package [50]. Gray-color squares represent non-detected values. Metabolites are grouped in amino acids and derivatives (AA), organic acids (OA), sugars and sugar alcohols (SS), and others (O).

**Figure 2:**
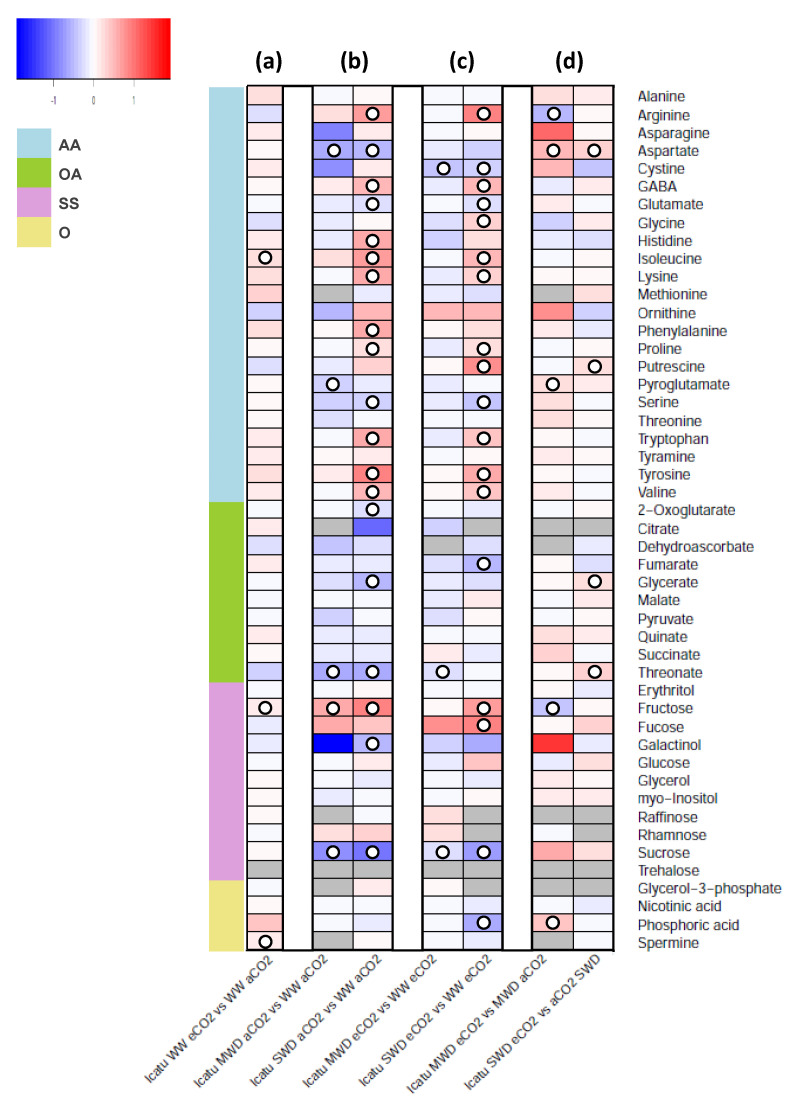
Heatmap representing the changes in relative levels of primary metabolites in leaves of *Coffea arabica* cv. Icatu (Icatu) grown under 380 (aCO_2_) or 700 (eCO_2_) ppm CO_2_, and three water availability levels (WW, well-watered; MWD, moderate water deficit; SWD, severe water deficit). Results indicate (**a**) single exposure to eCO_2_, (**b**) single exposure to each water deficit level under aCO_2_, (**c**) exposure to each water deficit level under eCO_2_, and (**d**) eCO_2_ effect within each water deficit level. Relative values (as means of three to six independent measurements) were normalized to the internal standard (ribitol) and the dry weight of the samples; false color imaging was performed on log_10_-transformed GC-TOF-MS metabolite data. Significant changes using one-way ANOVA are indicated as (○) for *p* < 0.05. Heatmaps and clustering were performed in R software [48,49] using the “gplots” package [50]. Gray-color squares represent non-detected values. Metabolites are grouped in amino acids and derivatives (AA), organic acids (OA), sugars and sugar alcohols (SS), and others (O).

The GC-TOF-MS analysis identified 48 primary metabolites in CL153 and Icatu leaves, including 23 amino acids and derivatives, 10 organic acids, 11 sugars, and 4 other metabolites (Figure 1 and Figure 2, Supplementary Tables S1 and S2).

In general, a larger number of significant changes in primary metabolite levels were observed in CL153 than in Icatu due to single exposure to eCO_2_ (WW plants), and MWD (both CO_2_ concentrations). However, under SWD, both genotypes showed a large (and similar) number of significant changes under both aCO_2_ or eCO_2_. Additionally, in CL153 SWD plants under eCO_2_, those changes reflected mostly a decrease in metabolite levels (as opposed to Icatu) (Figure 1c and Figure 2c). Lower metabolite levels were observed under eCO2 as compared with aCO_2_ in both MWD or SWD (Figure 1d), whereas Icatu revealed opposite patterns under eCO_2_, as compared with aCO_2_ (Figure 2d).

In CL153 plants, the effect of single exposure to eCO_2_ led to a significant increase in aspartate, glutamate, methionine, pyroglutamate, 2-oxoglutarate, citrate, quinate, and glucose (up to 5-fold) (Figure 1a). Additionally, exposure to single drought conditions caused a higher number of significant changes in primary metabolite levels (Figure 1b). Under MWD, CL153 plants grown under aCO_2_ revealed significant increases (up to 5-fold) in most amino acids (γ-aminobutyric acid (GABA), glutamate, histidine, isoleucine, lysine, pyroglutamate, threonine, tryptophan, tyramine, tyrosine, and valine). A similar response was observed under SWD with significant increases in amino acids (up to 11-fold), together with a significant increase in glucose (5-fold), and a significant decline in sucrose, citrate, and phosphoric acid. Interestingly, CL153 plants grown under eCO_2_ and subjected to MWD and SWD conditions (Figure 1c) showed a markedly different response than under WW conditions, with reduction of a large number of metabolites. These eCO_2_ plants also showed a different pattern under MWD and SWD as compared with their aCO_2_ counterparts, revealing significant declines in the levels of asparagine, aspartate, glycine, glutamate (only at SWD), histidine (only at MWD), proline (only at MWD), pyroglutamate, and serine. Nevertheless, under eCO_2_, significant increases in glucose (2-fold, in MWD), and in fructose (4-fold), trehalose (5-fold), tryptophan, (up to 7-fold), and valine (4-fold) were observed under SWD. Significant reductions were observed regarding pyruvate (under MWD), and in citrate, glycerate, and threonate under SWD.

Furthermore, a global reduction in relative metabolite abundance was observed when comparing eCO_2_ to aCO_2_ for each water deficit treatment in regards to amino acid levels, particularly in MWD (Figure 1d). Still, under the combined exposure to SWD and eCO_2_, only glutamate and pyroglutamate showed a significant decrease, whereas pyruvate and sucrose showed a significant increase (up to 4-fold).

Analysis of *C. arabica* cv. Icatu showed a different pattern of metabolite variation when compared with the *C. canephora* genotype CL153. In Icatu plants, the single eCO_2_ effect promoted significant changes in a low number of metabolites (in isoleucine, fructose, and spermine, up to 2-fold) (Figure 2a). Furthermore, in this genotype, only a few metabolites showed altered abundance under MWD, irrespective of the air CO_2_ concentration (Figure 2b). Most significant changes were observed in plants under SWD and aCO_2_ conditions (Figure 2b,c). Overall, these significant variations included an increase in arginine, fructose, GABA, histidine, isoleucine, lysine, phenylalanine, proline, tryptophan, tyrosine, and valine (up to 9-fold), and a significant decrease in 2-oxoglutarate, aspartate, galactinol, glutamate, glycerate, serine, sucrose, and threonate.

In opposition to what was observed in CL153, comparison between the combined effect of MWD and SWD in plants grown under eCO_2_ (Figure 2d) led to few significant changes in primary metabolite levels. These changes included an increase in aspartate (up to 4-fold), pyroglutamate (2-fold, in MWD under eCO_2_), putrescine, glycerate, threonate (2-fold, in SWD under eCO_2_), and a significant decrease in arginine and fructose (in MWD under eCO_2_).

**Figure 3:**
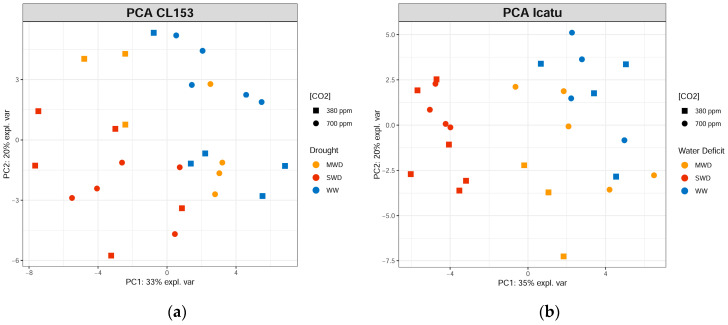
Principal component analysis (PCA) score plots of the primary metabolite profile of leaves of (**a**) *Coffea canephora* cv. Conilon Clone 153 (CL153) and (**b**) *C. arabica* cv. Icatu (Icatu), grown under 380 (aCO_2_) or 700 (eCO_2_) ppm CO_2_ and increasing water deficit severity; namely, well-watered (WW), mild water deficit (MWD), and severe water deficit (SWD). Plots performed in R software [48,49] using the “mixOmics” package [51].

Unsupervised principal component analysis (PCA) was applied to GC-TOF-MS relative primary metabolite data to identify major sources of variation in the data (Figure 3). The CL153 SWD plants clustered separately in relation to WW and MWD plants, whereas a distinction between CO_2_ concentration conditions could only be observed for MWD plants.

In Icatu plants (Figure 3b), a clear separation was observed for SWD plants along PC1, in relation to WW and MWD plants. In addition, as observed for CL153 plants, only MWD plants showed a clear distinction between plants grown under aCO_2_ and eCO_2_.

**Figure 4:**
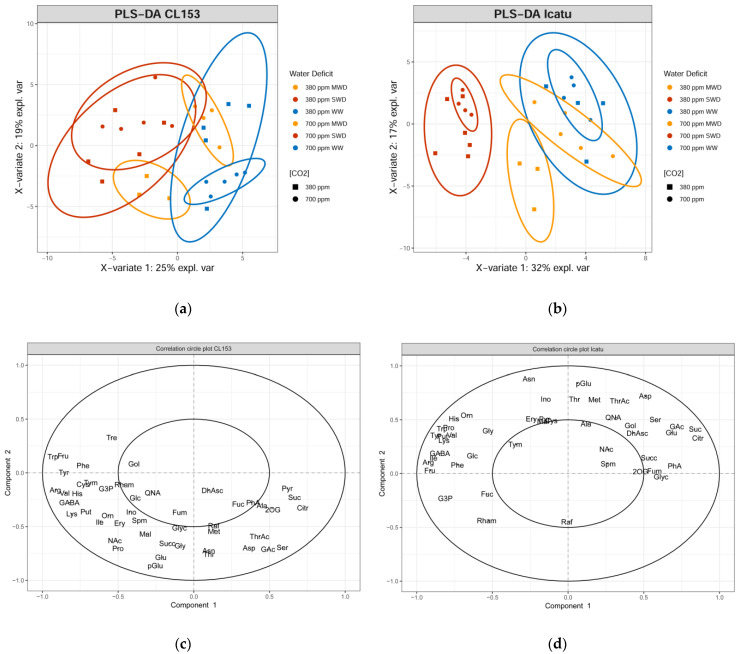
Partial least square discriminant analysis (PLS-DA) score and correlation circle plots of the primary metabolite profile of leaves of *Coffea canephora* cv. Conilon Clone 153 (CL153), (**a**,**c**), respectively; and of *C. arabica* cv. Icatu (Icatu), (**b**,**d**), respectively, grown under 380 (aCO_2_) or 700 (eCO_2_) ppm CO_2_ and increasing water deficit severity; namely, well-watered (WW), mild water deficit (MWD), and severe water deficit (SWD). Plots performed in R software [48,49] using the “mixOmics” package [51]. Abbreviations: 2-oxoglutarate (2OG), alanine (Ala), arginine (Arg), asparagine (Asn), aspartate (Asp), citrate (Citr), cystine (cys), dehydroascorbate (DhAsc), erythritol (Ery), fructose (Fru), fucose (Fuc), fumarate (Fum), galactinol (Gol), glucose (Glc), glutamate (Glu), glycerate (GAc), glycerol (Glyc), glycerol-3-phosphate (G3P), glycine (Gly), histidine (His), isoleucine (Ile), lysine (Lys), malate (Mal), methionine (Met), *myo*-Inositol (Ino), nicotinic acid (NAc), ornithine (Orn), phenylalanine (Phe), phosphoric acid (PhA), proline (Pro), putrescine (Put), pyroglutamate (pGlu), pyruvate (Pyr), quinate (QNA), raffinose (Raf), rhamnose (Rham), serine (Ser), spermine (Spm), succinate (Succ), sucrose (Suc), trehalose (Tre), threonate (ThrAc), threonine (Thr), tryptophan (Trp), tyramine (Tym), tyrosine (Tyr), valine (Val).

To further explore and identify potential stress responsive metabolites in these *Coffea* spp. genotypes, a supervised partial least square discrimination analysis (PLS-DA) was performed (Figure 4). As observed in the PCA, both genotypes showed a clear discrimination between WW and SWD plants, particularly in Icatu plants, regardless of CO_2_ concentration. PLS-DA further confirmed a clear distinction between MWD plants grown under different CO_2_ concentrations.

The score plots revealed a high number of highly correlated metabolites responsible for the cluster of SWD samples in the negative quadrants of component 1 for both genotypes. These metabolites include mostly amino acids and sugars that significantly increased in plants exposed to single and combined drought and eCO_2_ conditions, including the the branched chained amino acids (BCAAs) valine and isoleucine, arginine, GABA, lysine, phenylalanine, trehalose, tryptophan, and tyrosine for CL153 plants, and arginine, BCAAs, fructose, GABA, lysine, proline, tryptophan, and tyrosine for Icatu plants.

## 3. Discussion

GC-TOF-MS analysis allowed for the characterization of the primary metabolome of CL153 and Icatu leaves under single and combined exposure to increasing CO_2_ concentration (eCO_2_) and water deficit (MWD and SWD) conditions. Unsupervised exploratory PCA revealed differential metabolite responses to single and combined stresses; this effect was clearer in Icatu plants, and PLS-DA analyses allowed us to further identify stress-responsive metabolites whose levels significantly varied upon single and/or combined exposure to drought and eCO_2_.

The significant increase in CL153 alone of most amino acids upon single exposure to MWD indicated that Icatu was not affected by MWD, and thus, supports previous studies reporting its strong drought resilience [32]. However, under SWD, both genotypes showed relevant changes in the levels of most amino acids, although in Icatu these changes were relatively similar under both air CO_2_ concentrations. This is also in agreement with the reported drought resilience of this genotype under both CO_2_ concentrations [32]. The BCAAs isoleucine and valine significantly increased in Icatu under SWD at both air CO_2_ concentrations, and under MWD only at aCO_2_, whereas in CL153 an increase was only observed under SWD and eCO_2_. BCAAs are well-known to accumulate under abiotic stress conditions and play the role of osmolytes during osmotic stress [36,52,53]. BCAAs are also responsible for an alternative supply of electrons and respiratory substrates to the plant respiratory chain under stress conditions, ultimately contributing to drought stress tolerance in plants [54,55].

Lysine, a product of the aspartate pathway, also significantly increased in response to SWD in CL153 plants (only under aCO_2_), and Icatu (under aCO_2_ and eCO_2_). Lysine catabolism can channel electrons to the mitochondrial electron transport chain in addition to feeding the operation of the tricarboxylic acid (TCA) cycle under carbon starvation [54], as would be the case in these plants due to a strong reduction of photosynthetic C-assimilation under limited water availability [32].

Metabolites from the glutamate family pathway significantly increased in Icatu, including the osmolytes proline and GABA, under SWD irrespective of CO_2_ concentration. Osmolyte accumulation has an important role in abiotic stress responses; namely, maintenance of cell turgor via a decrease in the osmotic potential, protection against oxidative damage by decreasing the levels of reactive oxygen species (ROS), and maintenance of the cellular redox balance [41,42,43]. Accumulation of proline is considered an adaptive mechanism in drought-tolerant coffee genotypes. For instance, *C. canephora* clones (IC-2, IC-3, IC-4, IC-6, IC-8, and R-4) subjected to drought-stress revealed an accumulation of proline with increasing exposure to soil drying, followed by a decline upon re-watering [56]. Moreover, Silva et al. [57] showed that proline accumulation was higher in the leaves of a drought-tolerant clone of *C. canephora* than in a susceptible genotype. Proline levels were also found to increase in *C. arabica* cvs. Catuaí and Mundo Novo, *C. canephora* cv. Apoatã, and a graft of Mundo Novo shoot on Apoatã root in response to drought [58]. Arginine, another member of the glutamate family, showed a significant increase in response to the harshest water deficit level under both CO_2_ concentrations and in both genotypes. Increased levels of arginine were suggested to contribute to drought tolerance [59]. Arginine is a precursor of polyamines, molecules strongly associated with many biological processes including protection from osmotic stress [60]. TCA cycle-derived amino acids of the glutamate and aspartate families are central regulators of carbon and nitrogen metabolism [61]. In fact, a high level of glutamine synthetase activity has been found in the leaves of *C. arabica* cv. Catuaí during grain expansion [62], which has been linked to the capacity of coffee plants to assimilate nitrogen, a useful trait to improve coffee management in commercial orchards.

In Icatu, amino acids derived from the shikimate pathway (e.g., phenylalanine, tryptophan, and tyrosine) accumulated under single exposure to SWD under aCO_2_; tryptophan and tyrosine also increased in SWD under eCO_2_ conditions. The accumulation of these metabolites is a valuable source of carbon-skeletons for the phenylpropanoid pathway and further biosynthesis of secondary metabolites [63,64]. Tryptophan is reported to play an important role as an osmolyte, but also in stomatal regulation and reactive oxygen species (ROS) scavenging under drought conditions [65].

A significant increase in putrescine occurred in Icatu under the interaction of SWD and eCO_2_. Several drought experiments have shown a correlation between resistance to dehydration and the levels of putrescine—a plant growth regulator that helps to balance chlorophyll concentrations—and the accumulation of osmolytes and soluble phenolic compounds under drought conditions [66]. 

Osmoprotective sugars were also affected by drought and a significant increase was observed in fructose in Icatu and in glucose, trehalose, and sucrose in CL153 (particularly under SWD and eCO_2_ conditions), which might have contributed to dehydration tolerance [36,37].

The metabolite responses of each genotype to water deficit and eCO_2_, together with the PCA and PLS-DA analysis, showed that eCO_2_ clearly altered the impact of MWD in both genotypes, as shown by the overlap of the MWD under eCO_2_ cluster with WW controls. However, MWD plants under aCO_2_ did not show such proximity to WW controls. This observation is in line with the positive effect of eCO_2_ regarding the water deficit imposition reported earlier [28,32], mainly in Icatu plants. In addition, the clear separation of SWD plants from the WW and MWD clusters, particularly in Icatu plants, showed that the production of metabolites was greatly altered by SWD. Nevertheless, Icatu plants maintained (or increased) their metabolite levels under both CO_2_ concentrations (within MWD and SWD), contrasting with the decreases observed in CL153 under eCO_2_, which is in line with the high tolerance of Icatu plants to water deficit under both CO_2_ concentrations [32].

Physiological and biochemical studies demonstrated an absence of impact on Icatu plants in most parameters under SWD, irrespective of CO_2_ concentration, and a greater tolerance than CL153 under aCO_2_. This resilience of Icatu was associated with the preservation of photosynthetic performance (e.g., photosynthetic capacity, A_max_; photochemical efficiency of PSII, F_v_/F_m_) and the maintenance or reinforcement of several chloroplast components (e.g., cytochromes electron carriers, photosystems (PSs), and RuBisCO activities) [32,44]. Such intrinsic tolerance (reinforced under eCO_2_) was further associated with a wide abundance of proteins related to the photosynthetic apparatus (as part of photosystems, light harvesting complexes, protective cyclic electron flow, and RuBisCO activase), which was not observed in CL153 [32].

Limited photochemical energy use can generate ROS leading to damage to the photosynthetic apparatus (e.g., D1 protein, lipids, electron transport) [67,68]. Therefore, at the cellular level, drought tolerance is often associated with the activation of photoprotective and antioxidative mechanisms. This has previously been shown to be a key response in the acclimation of coffee plants to a wide variety of abiotic stresses, such as high irradiance [69,70], cold [71,72,73], and temperature [19,21,31]. Antioxidant enzymes coupled with strong lipid dynamics usually contribute to the maintenance of photosynthetic activity in coffee genotypes (including in Icatu). For instance, drought experiments using *C. canephora* plants of Clone 120 (drought-tolerant) and Clone 109 (drought-sensitive), subjected to repetitive cycles of drought conditions for 14 days, revealed an oxidative stress response on the sensitive clone, presumably leading to programmed cell death, while acclimation of the tolerant genotype seems to be related to antioxidant secondary metabolites and ABA response [74].

## 4. Materials and Methods

### 4.1. Plant Material and Growth Conditions

Plants of two cropped genotypes (Brazil) from the two main coffee-producing species, *Coffea canephora* Pierre ex A. Froehner cv. Conilon Clone 153 (CL153) and *C. arabica* L. cv. Icatu Vermelho (an introgressed cultivar resulting from a cross of *C. canephora* and *C. arabica* cv. Bourbon Vermelho, which was then further crossed with *C. arabica* cv. Mundo Novo) were used, as described in [32]. Briefly, plants were grown for seven years in 80 L pots in two walk-in growth chambers (EHHF 10000, ARALAB, Portugal), under controlled temperature conditions (25/20 °C, day/night, ±1 °C), irradiance (ca. 750 μmol m^−2^ s^−1^ at the upper part of the plant), relative humidity (70% ± 2%), photoperiod (12 h), and either ambient (aCO_2_, 380 ± 5 ppm) or elevated (eCO_2_, 700 ± 5 ppm) air CO_2_ concentration. Plants were grown without restriction of nutrients [14] or root growth, and with adequate water availability provided by watering the plants every two days.

### 4.2. Water Deficit Imposition

Within each chamber, plants were divided into three groups (*n* = 6 to 8) and maintained under well-irrigated conditions (WW), or exposed to moderate (MWD) and severe water deficit (SWD), as described in [32]. Water deficit was gradually imposed over two weeks by partially withholding irrigation (that is, by replacing only part of the amount of lost water per pot). Water availability levels were established as a function of leaf water potential at predawn (Ψ_pd_), as WW: Ψ_pd_ > −0.35 MPa; MWD −1.5 < Ψ_pd_ < 2.5 MPa; and SWD Ψ_pd_ < 3.5 MPa, corresponding to ca. 80% (WW), 25% (MWD), or 10% (SWD) of maximal pot water availability, respectively [75]. Ψ_pd_ was determined immediately after leaf excision in 5–6 plants per treatment, using a pressure chamber (Model 1000, PMS Instrument Co., Albany, OR, USA).

When the required Ψ_pd_ was reached for MWD or SWD conditions, pot moisture was maintained for another two weeks by adding adequate water amounts according to each watering treatment before sampling. Exceptionally, the Icatu 700-plants (eCO_2_) under MWD conditions were exposed to total water withholding in the last five days of the four-week period, in order to further force the reduction of Ψ_pd_ values, which did not shift below −0.6 MPa until that stage.

For metabolomic analysis, newly matured leaves from the upper third part (well illuminated) were collected, immediately frozen in liquid nitrogen, and stored at −80 °C.

### 4.3. GC-TOF-MS Primary Metabolite Profiling

Primary metabolites were extracted and derivatized following a well-established protocol [76]. Frozen leaves were ground to a fine powder with a mortar and pestle and under liquid nitrogen. A total of 50 mg fresh weight (FW) of finely homogenized leaf material was weighed into each 1.5 mL safe-lock polypropylene microfuge tube and 700 µL of ice-cold methanol and 30 μL of ribitol (0.2 mg mL^–1^ ribitol in water), as the internal standard, were added to each tube. Samples were vortex-mixed and incubated in a shaker (ThermoMixer, Eppendorf, Hamburg, Germany) for 15 min at 70 °C and 950 rpm, and subsequently centrifuged (12,000× *g*, 10 min, 20 °C). The supernatant was collected, mixed with 375 μL chloroform and 750 μL water, and vortex-mixed. After centrifugation (2200× *g*, 15 min, 20 °C), 150 mL of the polar aqueous/methanol phase was evaporated to dryness using a centrifugal concentrator for 3 h at 30 °C (Vacufuge Plus, Eppendorf, Hamburg, Germany), and stored at −80 °C. Dried polar extracts were derivatized with 40 μL of 20 mg mL^−1^ methoxyamine hydrochloride in pyridine, followed by 70 μL of *N*-methyl-*N*-trimethylsilyltrifluoroacetamide (TMS derivatization) and 20 μL mL^−1^ of a mixture of fatty acid methyl esters (FAMES). Biological variations were controlled by analyzing quality control (QC) standards; namely, FAMES internal standard markers and a QC standard solution of 41 pure reference compounds, throughout the analysis. After GC-TOF-MS analysis, samples were subsequently evaluated using AMDIS (Automated Mass Spectral Deconvolution and Identification System software, version 2.71). Primary metabolites were annotated using the TagFinder software [77] and a reference library of ambient mass spectra and retention indices from the Golm Metabolome Database (http://gmd.mpimp-golm.mpg.de/, accessed on 25 June 2021) [78,79]. Metadata information following minimum reporting standard guidelines of the Metabolomics Standard Initiative (MSI) can be found in Supplementary Table S3.

### 4.4. Experimental Design and Statistical Analyses

Plants from each coffee genotype were subjected to six treatment combinations, reflecting the single and combined exposure to two CO_2_ concentration (aCO_2_ or eCO_2_) and three levels of water availability (WW, MWD or SWD). The experiment was implemented using a completely randomized design, with six to eight plants per treatment in individual pots.

Statistical analyses were performed in R and R Studio software [48,49]. R Packages used to perform statistical analysis include “agricolae” [80], “gplots” [50], and “mixOmics” [51].

The relative abundance of primary metabolite levels was normalized to the internal standard (ribitol) and the fresh weight/dry weight ratio of the samples. Within each genotype, one-way ANOVA at a 95% confidence level was used to assess differences between treatments. Subsequently, fold-changes between conditions and the control for each metabolite were determined and Log_10_ transformed for heatmap plotting.

Principal component analysis (PCA) was applied using unit variance scaling, and to further maximize the discrimination between the sample groups, supervised partial least squares discriminant analysis (PLS-DA) was performed using the leave-one-out cross-validation embedded in the “mixOmics” package.

## 5. Conclusions

Overall, our metabolite profiling findings showed significant drought impacts on two widely traded coffee species. Notably, eCO_2_ clearly reduced metabolite changes under MWD in Icatu (but not in CL153), resulting in metabolic profiles close to those of their WW plants. These findings are relevant in terms of the coffee plant response to future climate change scenarios (particularly in regard to the role of eCO_2_), and also highlight the need for complementary and integrative studies, from ecophysiology to omics, to better clarify the role of specific metabolites in the resilience of this important tropical crop.

## Data Availability

The data presented in this study are available in article and Supplementary Materials.

## Supplementary Materials

The following are available online at https://www.mdpi.com/article/10.3390/metabo11070427/s1, Table S1: Fold changes in relative levels of primary metabolites in leaves of *Coffea canephora* cv. Conilon: Clone 153, Table S2: Fold changes in relative levels of primary metabolites in leaves of *Coffea arabica* cv. Icatu, Table S3: Metabolomics Standards Initiative compliant metadata.
